# Predictive and Experimental Immunogenicity of *Burkholderia* Collagen-like Protein 8-Derived Antigens

**DOI:** 10.3390/vaccines9111219

**Published:** 2021-10-20

**Authors:** Megan E. Grund, Eliza Kramarska, Soo Jeon Choi, Dudley H. McNitt, Christopher P. Klimko, Nathaniel O. Rill, Jennifer L. Dankmeyer, Jennifer L. Shoe, Melissa Hunter, David P. Fetterer, Zander M. Hedrick, Ivan Velez, Sergei S. Biryukov, Christopher K. Cote, Rita Berisio, Slawomir Lukomski

**Affiliations:** 1 Department of Microbiology, Immunology and Cell Biology, School of Medicine, West Virginia University, Morgantown, WV 26506, USA; meg0053@mix.wvu.edu (M.E.G.); sochoi@hsc.wvu.edu (S.J.C.); dudley.mcnitt@vumc.org (D.H.M.); 2 Institute of Biostructures and Bioimaging, National Research Council (CNR-IBB), 80134 Naples, Italy; eliza.kramarska@unicampania.it (E.K.); rita.berisio@cnr.it (R.B.); 3 Bacteriology Division, The United States Army Medical Research Institute of Infectious Diseases (USAMRIID), Frederick, MD 21702, USA; christopher.p.klimko2.civ@mail.mil (C.P.K.); nathaniel.o.rill.ctr@mail.mil (N.O.R.); jennifer.l.dankmeyer.civ@mail.mil (J.L.D.); jennifer.l.shoe.ctr@mail.mil (J.L.S.); melissa.hunter.ctr@mail.mil (M.H.); zander.m.hedrick.mil@mail.mil (Z.M.H.); ivan.velez15.mil@mail.mil (I.V.); sergei.s.biryukov.civ@mail.mil (S.S.B.); christopher.k.cote.civ@mail.mil (C.K.C.); 4 Biostatistics Division, The United States Army Medical Research Institute of Infectious Diseases (USAMRIID), Frederick, MD 21702, USA; david.p.fetterer.ctr@mail.mil

**Keywords:** vaccine, Bucl8, subunit vaccine, antigenicity, immunogenicity

## Abstract

*Burkholderia pseudomallei* is an infectious bacterium of clinical and biodefense concern, and is the causative agent of melioidosis. The mortality rate can reach up to 50% and affects 165,000 people per year; however, there is currently no vaccine available. In this study, we examine the antigen-specific immune response to a vaccine formulated with antigens derived from an outer membrane protein in *B. pseudomallei*, Bucl8. Here, we employed a number of bioinformatic tools to predict Bucl8-derived epitopes that are non-allergenic and non-toxic, but would elicit an immune response. From these data, we formulated a vaccine based on two extracellular components of Bucl8, the β-barrel loops and extended collagen and non-collagen domains. Outbred CD-1 mice were immunized with vaccine formulations—composed of recombinant proteins or conjugated synthetic peptides with adjuvant—to assess the antigen-specific immune responses in mouse sera and lymphoid organs. We found that mice vaccinated with either Bucl8-derived components generated a robust TH2-skewed antibody response when antigen was combined with the adjuvant AddaVax, while the TH1 response was limited. Mice immunized with synthetic loop peptides had a stronger, more consistent antibody response than recombinant protein antigens, based on higher IgG titers and recognition of bacteria. We then compared peptide-based vaccines in an established C57BL/6 inbred mouse model and observed a similar TH2-skewed response. The resulting formulations will be applied in future studies examining the protection of Bucl8-derived vaccines.

## 1. Introduction

*Burkholderia pseudomallei* is a Gram-negative bacterium that is the causative agent of melioidosis and is endemic in Southeast Asian countries and Australia, but is also present in Africa and Latin America [1]. A disease with a high morbidity and significant mortality rates, melioidosis is a rising global healthcare concern characterized by one of the highest disability-adjusted life years (DALYs) index among neglected tropical diseases [2]. *B. pseudomallei*, and its clonal derivative *Burkholderia mallei*, are recognized as concerning infectious and bioterror agents in the United States, and thus are classified as Tier one select agents by the Federal Select Agent program [3]. While melioidosis is not consider a healthcare or public health problem in the United States, there is an assumption that the bacterium may be present in soil and water in southwestern regions [4], and several linked cases were recently reported in patients thought to have been exposed to contaminated imported product(s) [5].

Without treatment, melioidosis has up to a 90% mortality rate. This issue is compounded by underreported cases due to non- or misdiagnosis. Infection commonly occurs from contact with contaminated soil/water, and therefore endemic regions are often rural agriculture communities that may not have access to appropriate healthcare. Symptoms drastically range from low grade fever to sepsis, and can be misinterpreted as other diseases such as tuberculosis, thus the patients would be given the wrong antibiotics. One of the major obstacles with treating an infection is *B. pseudomallei* has a high level of resistance to many antimicrobial drugs. The common treatment plan for patients with melioidosis involves a lengthy drug regimen that may not completely eradicate the bacteria [6]. *B. pseudomallei* can survive intracellularly, and therefore non-treatment or incomplete eradication may lead to the bacteria lying dormant in host cells, with reports of reemergence several decades later [7,8]. In short, treatment is not always effective.

Thus, a preventative approach, such as vaccination, is an appealing and logical combative measure against *Burkholderia*; however, there is no available vaccine against *B. pseudomallei* and/or *B. mallei* pathogens. As with other vaccines, emphasis has been placed on generating subunit vaccines due to their high safety profile compared to whole cell vaccines. Several antigens have been investigated as potential candidates, including Hcp1, a type VI secretion protein [9], FliC, a flagellin protein [10], and OmpW, an outer membrane barrel [11]. Capsular polysaccharide and lipopolysaccharide have also been used as single antigens or in combination with other antigens, such as Hcp1, to augment a response [9]. Current vaccine approaches include immunization with *Burkholderia*–derived outer membrane vesicles [12,13] and gold-nanoparticle conjugates [14,15] of promising antigen candidates: Hcp1, capsular polysaccharide, and OmpW [11]. Additionally, several live attenuated vaccine strategies have been examined with varying results [3,16,17]. Profiling the type of immune response from antigens, combinations of antigens, and establishing their safety profile is key to designing an effective vaccine.

*Burkholderia* collagen-like protein 8 (Bucl8) is a conserved protein found in *Burkholderia* species, with almost complete conservation between *B. pseudomallei* and *B. mallei* [18]. The protein is predicted to be a trimeric outer membrane component of a putative RND-like efflux pump [19]. Bucl8 consists of two main structural constituents: a periplasmic α- and outer-membrane β-barrels, and an extended extracellular portion composed of a collagen (CL) domain and a non-collagenous carboxyl terminal (Ct) region. A prior study determined the recombinant protein, produced in *Escherichia coli*, corresponding to Bucl8’s CL-Ct component forms a collagen triple helix [19]. Additionally, homology modelling has predicted two main surface-exposed structures that have the potential to be targeted for a subunit vaccine: the β-barrel-loops L1 and L2, and the extended extracellular CL-Ct portion [20].

In this study, we use predictive software to determine the antigenicity, allergenicity, and toxicity of the surface-exposed components of Bucl8. From these predictions, we formulated vaccines using two types of antigens: recombinant proteins based on the CL-Ct regions and synthetic conjugated peptides based on the β-barrel-loops. In addition, we compared two types of adjuvants, AddaVax and Quil-A, and two peptide conjugates with CRM_197_ (Cross-reacting material 197) or KLH (Keyhole limpet hemocyanin). For the vaccine model, we used CD-1 mice, which are a heterogeneous outbred strain that better models the diversity within human population than inbred models and also provides a robust model for vaccines [21]. We used a combination of different immunoassays to assess the strength and type of humoral response for the different vaccine formulations. Because CD-1 mice are not commonly used as a model in the melioidosis field, we also assessed and compared the immune responses to vaccine formulations in inbred C57BL/6 mice. We conclude that formulation containing mixed peptide conjugates provides the strongest, most robust response and will be used in further studies to assess level of protection.

## 2. Materials and Methods

### 2.1. Bacterial Strains and Growth

BSL2 *Burkholderia pseudomallei* strain Bp82, an avirulent Δ*purM* mutant of strain 1026b, which is exempt from the Select Agents list, as well as Bp82Δ*bucl8* mutant [19] were used to assess antibody-binding. Bacteria were routinely grown in Luria broth-Miller (LBM) with shaking and on Luria agar (LA) solid medium at 37 °C.

### 2.2. Animal Care and Use

All the CD-1 mice experiments were approved by the West Virginia University Institutional Animal Care and Use Committees (WVU-IACUC protocol 1804013711.2) and performed in accordance of National Institutes of Health Guide for the care and use of laboratory animals. For studies with CD-1 IGS strain (Charles River Laboratories), equal number of 5–6-week-old female and male mice were used, and experiment was repeated.

C57BL/6 mice experiments were approved by the USAMRIID IACUC and performed in accordance of National Institutes of Health Guide for the care and use of laboratory animals. For studies with the C57BL/6 strain (Charles River Laboratories, Frederick, MD, USA), 7–9-week-old (at time of vaccination) female mice were used.

### 2.3. Antigenicity Prediction

Antigenicity prediction was performed to determine the overall possible role of Bucl8 regions and epitopes in initiating an immune response. Consensus antigenicity predictions were performed using Vaxijen [22] and Vaxign-2 tools [23]. These tools base their algorithms on principal amino acid properties of a protein sequence. The tool BepiPred2 (http://www.cbs.dtu.dk/services/BepiPred/, 1 August 2021) [24] was used to determine the probability of the presence of linear B cell epitopes in the Bucl8 sequence. BepiPred is based on a random forest algorithm trained on epitopes annotated from antibody-antigen protein structures. Structure based epitope prediction was performed using ElliPro [25] and Discotope [26], starting from the homology model of Bucl8 (residues 84–505) [20]. Discotope identifies discontinuous B cell epitopes, i.e., epitopes whose residues are distantly placed in the sequence albeit close in space in the three-dimensional structure of the protein antigen.

T-cell epitopes are presented on the surface of an antigen presenting cell (APC), where they are bound to major histocompatibility (MHC) molecules in order to induce an immune response [27]. MHC class II binding predictions were computed using the Immune Epitope Database (IEDB) tools (http://tools.iedb.org/mhcii/, 1 Augest 2021) [28]. Predicted affinities of antigens to MHCII were computed as IC50 (nM). Peptides with IC50 values <50 nM are considered high affinity binders, <500 nM intermediate affinity and <5000 nM low affinity.

### 2.4. Analysis of T Cell Epitope Allergenicity and Toxicity

Allergenicity was computed using AllergenFP v.1.0 [29] and AllerTOP v2.0 [30] servers, which classify amino acids in the protein sequences using five E-descriptors describing amino acid hydrophobicity, molecular size, helix-forming propensity, relative abundance of amino acids, and β-strand forming propensity. Proteins are classified by k-nearest neighbour algorithm (kNN, k = 1) based on training set containing 2427 known allergens from different species and 2427 non-allergens.

Toxicity was computed with the ToxinPred (August 2021; ToxinPred (osdd.net)) protein scanning tool, based on machine learning techniques and quantitative matrix through the recognition of residues detected in toxins [31].

### 2.5. Vaccination Formulation

Vaccines were formulated with antigens derived from Bucl8 that were predicted to be extracellular in homology models [20]. Two main antigen types were tested: (i) recombinant proteins rBucl8-CL-Ct and rBucl8-Ct and (ii) synthetic peptide-conjugates pepL1 and pepL2. Recombinant proteins were purified via previously described methods [19]. Briefly, the CL-Ct- and Ct-encoding sections of Bucl8 were cloned into the *E. coli* vector pQE30 with N-terminal 6xHis-tag. Recombinant polypeptides were affinity-purified on HisPur^TM^ Cobalt Resins (Thermo Scientific) and protein purity and integrity confirmed by SDS-PAGE. PepL1 and pepL2 were synthesized by WatsonBio and conjugated either to CRM_197_ (Cross-reacting material 197) or KLH (Keyhole limpet hemocyanin). Additionally, we tested a Mix group of pepL1- and pepL2-CRM_197_ conjugates to assess synergistic effects. Antigens were combined with either AddaVax or Quil-A (InvivoGen, San Diego, CA, USA) as indicated. Control groups included mice injected with PBS or adjuvant only.

### 2.6. Immunization Protocol

Mice were immunized subcutaneously three times 21 days apart with antigen-adjuvant formulations, as described [32]. Each immunization consisted of 25 µg of each antigen in a 100 µL of pyrogen-free saline mixed with an equal volume of the adjuvant; for the Mix pepL1/pepL2 group, mice were immunized with 25 µg of each antigen. Blood, spleens, and lymph nodes were harvested either 14 (CD-1 mice) or 27 days (C57BL/6) after the final booster to evaluate antibody types and titers, and cytokine responses.

### 2.7. ELISpot

Splenocytes were used to assess the number of antigen-specific B cells and interferon gamma (IFN-γ) production by ELISpot assays. Splenocytes were prepared as before [33]. Excised spleens were disaggregated in RPMI 1640 medium (ThermoFisher, Grand Island, NY, USA); red cells were lysed with ammonium-chloride-potassium (ACK) buffer (BioWhittaker, Walkersville, MD, USA). The extracts were diluted with RPMI 1640 medium and splenocytes were pelleted at 335 × g for 10 min. Cells were then re-suspended in CTL-Test Medium (Serum-free) (ThermoFisher, Grand Island, NY, USA) supplemented with L-glutamine.

To enumerate the antigen-specific B cells, 10^6^ of splenic cells/100 µL in culture medium were seeded in 96-well multiscreen filter plate (Millipore, Burlington, MA, USA) coated with 1 µg of non-conjugated peptide antigens (pepL1 and/or pepL2). Plates were incubated in 5% CO_2_, at 37 °C for 4 h, then washed with PBS/0.05% Tween20. 100 µL of secondary goat anti-mouse IgG antibody (Southern Biotech, Birmingham, AL, USA) in PBS/0.05% Tween20/1% BSA was added to wells and incubated at 4 °C overnight. Following washes with PBS-Tween, immune complexes were detected with a chromogenic substrate (Sigmafast BCIP/NCBI tablets) until spots had developed.

To assess IFN-γ production, Mouse IFN-γ Single-Color ELISPOT kit by ImmunoSpot^®^ (#mIFNγ-1M/2) was used. Wells were coated with anti-mouse IFN-γ capture mAb at 4 °C overnight and then washed with PBS. 100 µL of pepL1 or pepL2 (5 µg/mL) in CTL medium with 1% L-Glutamine was added to each well and incubated at 37 °C, 9% CO_2_ for 15 min. Splenocytes in CTL medium with 1% L-Glutamine were seeded at 4 × 10^5^ cells per well for peptide stimulation at 37 °C, 9% CO_2_ for 24 h. Splenocytes were removed and wells were washed with PBS and then with PBS/0.05% Tween. Biotinylated anti-mouse IFN-γ mAb was added to the wells and incubated at room temperature (RT) for 2 h. Following washes with PBS-Tween, immune complexes were detected by incubation with streptavidin-AP solution at RT for 30 min and then with development reagents according to manufacturer recommendations. Spots were scanned and analyzed using an automated ELISPOT reader (CTL-Immunospot S6 Analyzer, CTL, OH, USA). The splenocyte response was assessed as spot forming cells (SFC), adjusted to 10^6^ cells per well, which was automatically calculated by the ImmunoSpot^®^ Software for each stimulation condition and the medium only control.

### 2.8. Analysis of Antibody Responses by ELISA

Antigen-specific IgG antibody responses in CD-1 mice were measured by indirect ELISA. Wells were coated with 1 µg of each antigen (rBucl8-CL-Ct or rBucl8-Ct, and non-conjugated pepL1 and/or pepL2) in bicarbonate buffer for two hours at room temperature, then blocked overnight at 4 °C with 1% bovine serum albumin (BSA) in Tris-buffered saline (TBS). Sera from mice immunized with antigen/adjuvant (rBucl8-CL-Ct, rBucl8-Ct, pepL1-CRM_197_, pepL2-CRM_197_) were diluted 1:50 in TBS, added to wells, and incubated for two hours at 37 °C. Antigen-bound total IgG was detected with goat anti-mouse IgG AP-conjugate (Southern Biotech) and alkaline-phosphate (AP) substrate (PNPP; Thermo Scientific). Immunoreactivity was read at OD 405 nm and treatment groups were compared via a One-way ANOVA. Seroconversion in mice immunized with antigen/adjuvant combination was compared to that recorded in control mice treated with adjuvant (Quil-A and AddaVax) only. IgG subclass titers were measured for immune sera by diluting two-fold from a starting dilution of 1:50 in 1% BSA/TBS. IgG-subclass-specific goat anti-mouse IgG, IgG1, IgG2a, IgG2b in 1%BSA/TBS were used as secondary antibodies (Southern Biotech; 1:1000).

Total IgG titers from vaccinated C57BL/6 mice were determined by an ELISA performed in wells coated with non-conjugated peptides pepL1 or pepL2 diluted in 0.1 M carbonate buffer, pH 9.5, to a concentration of 2 µg/mL overnight at 4 °C. The wells were washed with 1X wash buffer (1× PBS, 0.05% Tween 20) and incubated with blocking buffer (1% Casein in PBS, Pierce/FisherScientific) for 30 min at 37 °C. After washing, ten twofold serial dilutions of mouse sera in 1x PBS, 0.25% casein made in triplicate wells were incubated for 1 h at 37 °C, then, were washed and reacted with secondary (Goat anti-mouse IgG- HRP conjugate; Southern Biotech; 1:5000) for 30 min at 37 °C. After the wells were washed, then, buffered hydrogen peroxide and 3,3′,5,5′-tetramethylbenzidine solution (Pierce, ThermoFisher) was added to each well and plates were incubated for 20 min at 37 °C. Next, reaction was stopped with 2 N sulfuric acid, and the amount of bound antibody was determined colorimetrically at 450 nm with a reference filter (570 nm) using a Biotek ELx808 plate reader (BioTek U.S., Winooski, VT, USA). The results are reported as the reciprocal of the highest dilution giving a mean OD of at least 0.1 (which was at least twice the background) ± 1 SD.

### 2.9. Surface Recognition of Bucl8 Antigen on Bp82 Cells

Polyclonal immune sera from CD-1 mice were pre-absorbed with the Bucl8-lacking mutant cells of Bp82 [19] to diminish a cross binding to whole bacterial cells. For the ELISA, wells were coated with ~10^4^ cells, either Bp82 or Bp82Δ*bucl8-fusE*, and incubated overnight at 4 °C. Wells were washed with 0.05% Tween-20/PBS and blocked with 1% BSA in 0.05% Tween-20/PBS at 37 °C for 2 h. Assay was completed as above.

To assess whole-cell binding by flow cytometry, Bp82 or Bp82Δ*bucl8-fusE* bacteria were grown from an overnight liquid culture to OD 0.4 and ~10^7^ cells were pelleted. Cells were washed with FACs buffer (PBS + 5% LBM) and then resuspended in 500 μL of cold FACs buffer. Absorbed immune sera were added to cell suspension at a dilution of 1:500 and incubated on ice for 30 min. Cells were washed and resuspended in solution with goat anti-mouse IgG Alexa 568 conjugate (Invitrogen) diluted 1:300, then incubated on ice for 30 min. Cells were washed and resuspended in 0.4% paraformaldehyde overnight at 4 °C. For analysis, cells were washed, resuspended in FACs buffer, and analyzed on a BD LSRFortessa flow cytometer.

### 2.10. Statistics

Statistical tests (Student’s *t*-test, One-Way ANOVA, Kruskal-Wallis) and post-hoc tests were performed using Graphpad Prism 6 software. Treatment groups were compared using One-way ANOVA and Tukey’s multiple comparison test, except for the titers. Titers were compared using a Kruskal-Wallis test with Dunn’s post-hoc test. For comparisons between only two groups, an unpaired Student’s *t*-test was used. Analysis between male and female mouse groups determined there were no statistical differences. Technical replicates were completed in triplicate.

## 3. Results

### 3.1. Prediction of Antigenic Epitopes Based on Sequence and Structure of Bucl8

**Figure 1:**
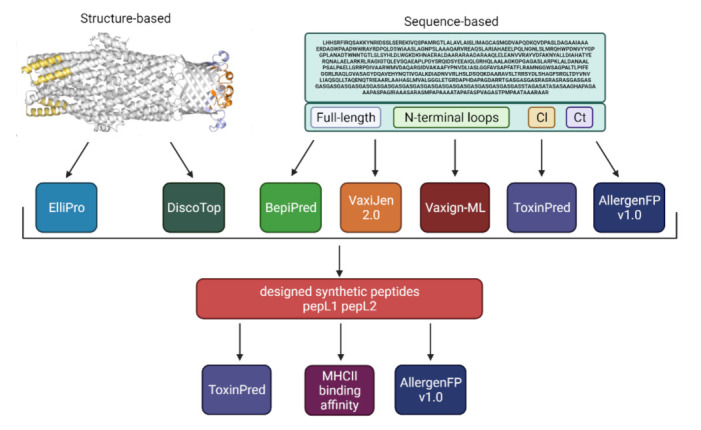
Schematic approach for using bioinformatic tools to predict immunogenic epitopes and their antigenicity, allergenicity, and toxicity.

We have recently homology modelled the structure of the *B. pseudomallei* outer-membrane protein Bucl8 and identified extracellular components as potential vaccine targets [20]. Here, we employed a number of bioinformatic tools to predict immunogenic epitopes, then, tested those antigens experimentally. Antigen prediction was performed on the whole Bucl8 sequence and specific domains, including the N-terminus, periplasmic loops, and CL and Ct sequence, using Vaxijen and Vaxign-ML tools (Figure 1 and Figure S1, Table 1). Overall, both software predicted Bucl8 to be a strong antigen, with high antigenicity scores according to Vaxijen (0.74) and Vaxign-ML (90.9%) (Table 1). Individual domains show elevated antigenicity, with the lowest value computed for the Ct domain. AllergenFP v1.0 and ToxinPred were used to assess the allergenicity and toxicity, respectively. All three Bucl8 domains are classified as non-toxic. In addition, all but the Ct domain are expected to be non-allergenic (Table 1).

**Figure 2:**
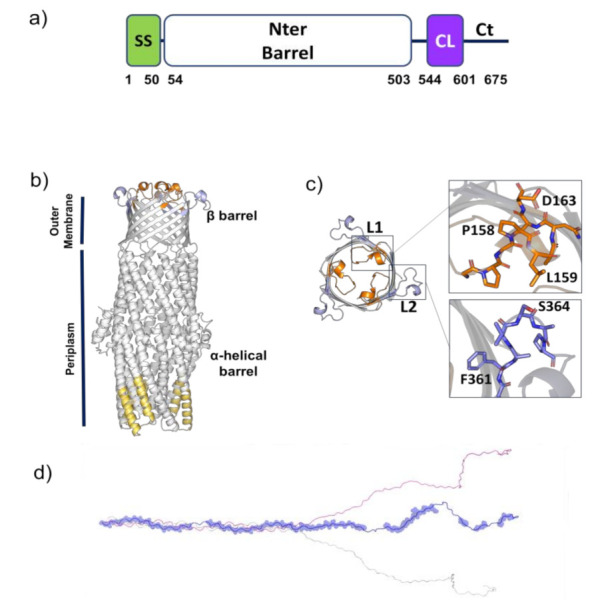
A structural view of Bucl8 (**a**) Domain borders of Bucl8. Numbers indicate position in amino acid sequence. SS, signal sequence; Nter, N-terminal; CL, collagen-like; Ct, C-terminal. (**b**) Cartoon representation of the homology model of Bucl8 N-terminal barrel domain, including a periplasmic α-helical barrel domain and a transmembrane β-barrel domain. Predicted structure-based antigens are shown in yellow on the α-helical barrel domain, and in orange and light blue on the β-barrel domain. (**c**) Top view of the transmembrane β-barrel domain of Bucl8. The two insets show enlargements of the L1 (orange) and L2 (light blue) peptides. (**d**) Cartoon representation of the model of the CL-Ct region of Bucl8. BepiPred predicted sequence-based antigens are drawn in one monomer as transparent spheres.

B cell epitopes can be classified as linear, made of single continuous stretch of amino acids within a protein sequence or conformational/discontinuous, where residues are distantly separated in the sequence and brought into physical proximity by protein folding. Sequence-based predictions of linear B cell epitopes were performed using BepiPred tool, with a threshold of 0.6 [24]. This analysis has highlighted that B cell epitopes exist in all three domains of the protein (Nter, CL, Ct, Table 2, Figure 2a and Figure S1). Given the well-known correlation between antigenicity, solvent accessibility, and flexibility of antigenic regions in proteins [34], the knowledge of the three-dimensional structure of an antigen helps in the reliability of antigen predictions. A reliable homology model, including residues 84-505, is readily available, given the significant sequence identity of Bucl8 with VceC efflux pump of *Vibrio cholerae* (PDB code 1yc9, seqid 35%) [35]. This model was used for structure-based epitope predictions, using the software ElliPro [25] and Discotope [26].

Antigen prediction with ElliPro detected the three strongest B cell epitope peptides (score > 0.7) of the N-terminus (Figure 2b, Table S1). Of these, peptide 3 is located on the α-helical barrel spanning the periplasm, whereas peptides 1 and 2 correspond to two loops (L1 and L2, respectively) located on the outer membrane β-barrel structure of Bucl8, and therefore accessible to antibodies (Table S1, Figure 2b,c). Discotope analysis also identified loop L1 as the best discontinuous epitope (Figure S2). In addition to the peptides, the extracellular CL-Ct domains were predicted by BepiPred to have targetable epitopes (Figure 2d). These domains are attractive as they are predicted to form a stalk structure that extends away from the cell surface, and thus more accessible to antibody binding.

From these predictions, we designed a synthetic peptide for each loop, designated pepL1 and pepL2, for subsequent experiments. These peptides are predicted to be non-toxic and non-allergenic (Table 3). They possess high MCHII-binding propensities, which is a prerequisite for human T cell immunogenicity (Table S2). In particular, pepL1 was predicted to hold high MCHII-binding affinity (IC50 < 50 nM, see methods). Lower MCHII-binding affinity is predicted for pepL2 (Table S2).

### 3.2. Generation of Antigen-Specific Antibodies from a Bucl8-Derived Vaccine

**Figure 3:**
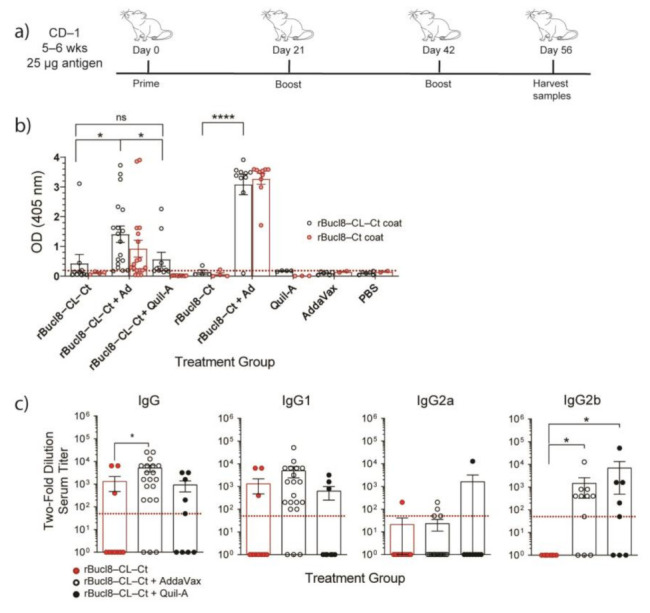
Immunogenicity of recombinant Bucl8-derived antigens. (**a**) Vaccine schedule. (**b**) Antigen-specific IgG response. Sera (1:50 dil) from CD-1 mice immunized with recombinant proteins rBucl8-CL-Ct or rBucl8-Ct, with and without adjuvant (AddaVax or Quil-A) were screened for total antigen-specific IgG level by ELISA. Wells were coated with either rBucl8-CL-Ct (black circle) or rBucl8-Ct (red circle). One-way ANOVA with Tukey’s post-hoc. Red line represents background OD. (**c**) Antigen-specific IgG subclasses. Sera obtained from rBucl8-CL-Ct treatment groups were analyzed, as indicated in the legend. Antibody titers were determined following two-fold dilution, starting at 1:50, until the OD_405_ was less than two-times the OD_405_ of BSA control. Red dotted line represents lowest positive titer (1:50). SEM error bars. Kruskal Wallis with Dunn’s post-hoc test * *p* < 0.05, **** *p* < 0.0001. ns; not significant.

Based on these predictions, we generated two types of immunogens to test for antigenicity: (i) 6xHis-tagged recombinant proteins rBucl8-CL-Ct and rBucl8-Ct, including the extended extracellular regions of Bucl8 (residues 545–675 and 601–675 for rBucl8-CL-Ct and rBucl8-Ct, respectively, Figure 2a,c) (ii) synthetic peptide-conjugates of the predicted pepL1 and pepL2. In an outbred murine model, we subcutaneously immunized female and male CD-1 mice with a vaccine containing antigen and adjuvant listed in Table 4. We adopted a vaccine schedule from a previous study [32], consisting of three immunizations 21 days apart, followed by specimen collection and processing 14 days after the last booster (Figure 3a).

Recombinant proteins were tested with and without adjuvants, either AddaVax, a squalene-based oil-emulsion, or Quil-A, a saponin-based adjuvant, both previously reported to help elicit a balanced TH1/TH2 immune response [36,37]. Combination of AddaVax with either recombinant protein showed enhanced antigen-specific antibody responses compared to the non-adjuvanted formulation or recombinant protein alone (Figure 3b). The formulation of rBucl8-CL-Ct and Quil-A showed a significantly lower antigen-specific response compared to the rBucl8-CL-Ct with AddaVax group, and therefore only vaccine formulations adjuvanted with AddaVax were used in following experiments. Additionally, sera from mice immunized with rBucl8-CL-Ct also recognized rBucl8-Ct protein, indicating mice will elicit a robust polyclonal response against the extended extracellular section of Bucl8, while immunized with this vaccine formulation.

Titers were completed to assess levels of antigen-specific IgG to compare adjuvanted and non-adjuvanted groups. rBucl8-CL-Ct + AddaVax has increased titers and number of responders compared to non-adjuvanted and Quil-A groups. Further analysis of IgG subclasses (Figure 3c), including IgG1, IgG2a, and IgG2b, determined that immunization with rBucl8-CL-Ct also generates higher IgG1 titers of antigen-specific antibodies when adjuvanted with AddaVax, compared with Quil-A formulation. Immunization with rBucl8-CL-Ct alone did not stimulate strong IgG2a/b response, and when adjuvanted the response was predominantly IgG2b. Additionally, we performed an ELIspot analysis to ascertain the number of antigen-specific B cells in the spleen, representing the systemic response (data not shown). Mice immunized with recombinant protein and AddaVax elicited a low number of antigen-specific B cells, which is contrary to the elicited antibody titers. Mice immunized with only adjuvant or PBS did not have an antigen-specific antibody or B-cell response.

**Figure 4:**
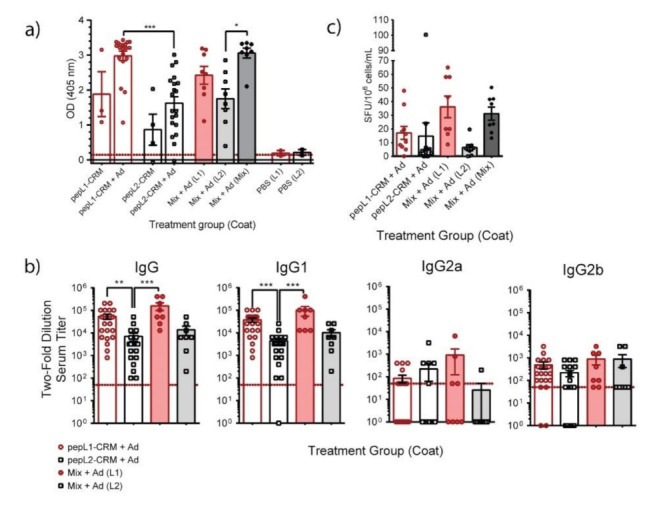
Immunogenicity of Bucl8-derived synthetic peptide antigens. (**a**) Antigen-specific IgG response. Sera (1:50 dil) from CD-1 mice immunized with synthetic peptide conjugates pepL1-CRM_197_, pepL2-CRM_197_, or Mix-CRM_197_, and with or without adjuvant AddaVax were screened for total antigen-specific IgG level by ELISA. Wells were coated with the corresponding non-conjugated peptides. Red line represents OD_405_ of BSA negative control. One-way ANOVA with Tukey’s multiple comparison test. (**b**) Antigen-specific IgG subclasses. Sera obtained from pepL1-CRM_197_, pepL2-CRM_197_, or Mix-CRM_197_ treatment groups were analyzed, as indicated in the legend. Antibody titers were determined following two-fold dilution, starting at 1:50, until the OD_405_ was less than two-times the OD_405_ of BSA control. Red dotted line represents lowest positive titer (1:50). Kruskal Wallis with Dunn’s post-hoc test. * *p* < 0.05, ** *p* < 0.01, *** *p* < 0.001. (**c**) Quantification of antigen-specific B cells in splenocytes. Homogenized splenocytes from immunized mice were added to wells coated with indicated antigen in parentheses. Spot forming units (SFU) were visually counted and counts were adjusted to the number of B cells determined by flow cytometry.

We next tested vaccine formulations containing two synthetic peptides, designated pepL1 and pepL2, derived from surface exposed loops (Figure 2b,c). Each peptide was conjugated to either genetically inactivated diphtheria toxoid CRM_197_ or traditionally used KLH. Initial experiments determined that KLH was inferior to CRM_197_ at producing antigen-specific antibodies (Figure S3); therefore, peptides conjugated to CRM_197_ combined with AddaVax were used in remaining immunizations. Both peptide immunogens elicited antigen-specific antibodies when screened by an indirect ELISA, which was not detected in PBS controls (Figure 4a). All mice immunized with pepL1, with or without adjuvant, produced antigen-specific total IgG response. PepL2-immunized mice showed a similar trend, although the level of response was significantly lower compared to pepL1 group. When mice were vaccinated with both antigens simultaneously, designated as the Mix group, we detected antigen-specific IgG reacted with pepL1 and pepL2; immunoreactivity in wells coated with both peptides was significantly higher than in wells coated with pepL2. IgG1 subclass of adjuvanted pepL1-CRM_197_, pepL2-CRM_197_, and Mix treatment groups had high comparable titers (Figure 4b). The pepL2-CRM_197_ treatment group had significantly lower total IgG and IgG1 titers compared to pepL1-CRM_197_ and Mix. Groups vaccinated with either pepL1, pepL2, or Mix with AddaVax generated a similar, low IgG2b response. In contrast, only the IgG2a response varied and not all the mice responded. Additionally, we found mice immunized with peptide generated antigen-specific B cells, which was determined by an ELISpot (Figure 4c).

**Figure 5:**
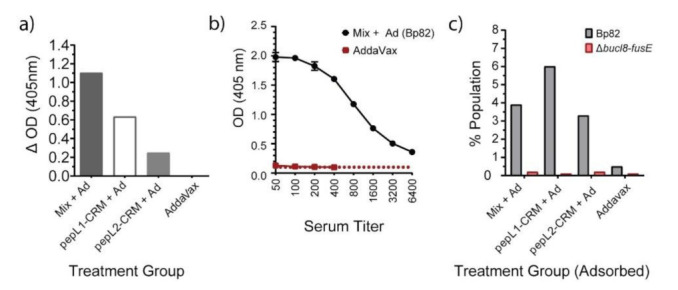
Antigen-specific IgG binding to *Burkholderia*. (**a**) Immune mouse sera recognize target bacteria by whole-cell ELISA. Wells were coated with ~10^4^ cells, either Bp82 or Bp82Δ*bucl8-fusE*. Sera from mice immunized with peptide antigens (pepL1, pepL2, Mix) were pre-adsorbed with Bp82Δ*bucl8-fusE* mutant cells before completing the ELISA, then added to wells at a 1:50 dilution. Immunoreactivity obtained in wells coated with mutant cells was subtracted from the corresponding wells coated with Bp82. α-IgG secondary, 1:1000 dilution. (**b**) Concentration-dependent antigen recognition. Serial two-fold dilution of serum obtained from the Mix + AddaVax was tested against whole cell Bp82. Titers were completed as described in Figure 4, using adsorbed sera. (**c**) Immune mouse sera recognize target bacteria by flow cytometry analysis. Pre-adsorbed sera were incubated with either bacteria, labeled.

We next assessed whether the antibodies from peptide treatment groups could recognize antigen on whole B. pseudomallei cells by ELISA and flow cytometry (Figure 5). To decrease cross-binding background, we used mouse immune sera pre-absorbed with a Bp82-mutant lacking the bucl8-associated locus, designated Δbucl8-fusE [19]. We assesed antigen recognition by comparing whole-cell antibody binding to Bp82 and Bp82Δbucl8-fusE (Figure 4). Data are represented as the difference between the binding signals, showing that sera from mice immunized with adjuvanted Mix, pepL1-CRM_197_, and pepL2-CRM_197_ all recognized the Bucl8 antigen on the B. pseudomallei cell (Figure 5a). Titration of the Mix + AddaVax sera resulted in an archetypical concentration-dependent binding curve, while sera from mice treated with AddaVax remained constant (Figure 5b). We confirmed these results with a similar whole-cell binding assay using flow cytometry. Immunized sera bound to Bp82, but not the mutant cells lacking Bucl8 (Figure 5c). The negative control showed a slightly increased level of binding to Bp82 compared to the mutant, indicating there is some non-specific background. Sera from mice treated with PBS and AddaVax did not produce any SFUs (not shown).

**Figure 6:**
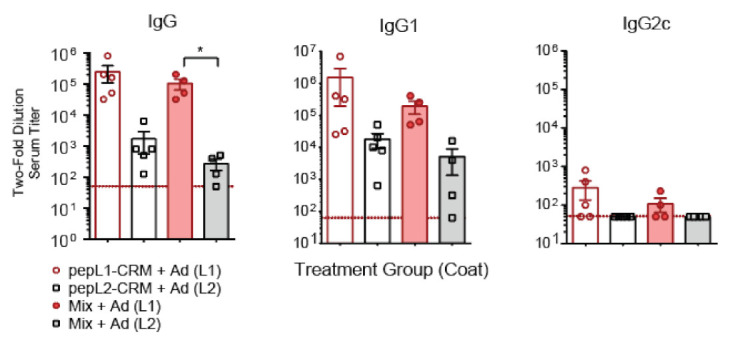
Antigen-specific immune response to pepL1 and pepL2-based vaccine in C57BL/6 mice. Serum titers of IgG subclasses (IgG, IgG1, IgG2c) from mice immunized with pepL1-CRM, pepL2-CRM, or Mix. Titers were measured by diluting sera 1:50 and then diluting two-fold; data is shown as the reciprocal titer. Red line indicates minimum positive titer (1:50). Kruskal-Wallis with Dunn’s post-hoc test. * *p* < 0.05.

Because CD-1 mice are not as commonly used as inbred models, we wanted to compare the humoral response in CD-1 mice to C57BL/6 mice. Mice were immunized with peptide loop formulations and assessed for antigen-specific response. We found a similar trend in C57BL/6 mice when comparing antibody titers. Immunization with either peptide resulted in high IgG and IgG1 titers, but little IgG2c response (Figure 6). For both mouse models, we observed lower responses to pepL2 than pepL1. However, mixing the two peptides did not appear to deter either response.

## 4. Discussion

There is currently no licensed *Burkholderia* vaccine; however, due to the pathogens’ intrinsic antimicrobial resistance, the mortality rate associated with the disease, and biothreat concerns, there is a pressing need for one. Numerous vaccine candidates have been investigated, ranging from live-attenuated bacteria [3] to subunit acellular vaccines [9,11,32,38,39], all with varying survival and protection efficacies. There is growing support for multivalent vaccines where the combined proteins and polysaccharides elicit effective and simultaneous humoral and cell-mediated responses [15,40]. Additionally, outer membrane proteins like Bucl8 have been targeted for vaccines because their surface-exposed components elicit recognition by the immune system, can be conserved, and expression is often vital for bacteria. Notable examples of OMPs targeted in *Burkholderia* include OmpW [11,15,41], OmpA [42,43], and Omp85 [32]. Here, we predict and then assess experimentally several novel antigens derived from the recently identified outer membrane protein found in *Burkholderia*, Bucl8.

*Burkholderia* are intracellular bacteria, which classically would be effectively targeted by a TH1-like response; however, there remains a gap in knowledge as to what type of immune response protects against *Burkholderia* infections. Uniform correlates of protection have not been defined for *Burkholderia* spp. and the literature is inconclusive whether an antigen-elicited humoral or cell-mediated response is more beneficial and protective. A majority of studies conclude that antibodies, driven by a TH2-like response, have a functional role in protection from infection [44,45], while TH1/TH17 cytokines such as IFN-γ, TNF-α, IL-17, and IL-6, have also been correlated with protection or bacterial killing [44,46]. Therefore, a vaccine that elicits both a simultaneous TH1/TH17 and TH2 immune response would be ideal. Our selection of adjuvants, AddaVax and Quil-A, reflect this concept and have been shown to elicit a balanced immune response in previous studies [36,47]. Here, we used the IgG subclass titers IgG1 and IgG2a/b, as indicators for a TH2 and TH1 response, respectively.

The recombinant proteins rBucl8-CL-Ct and rBucl8-Ct embed regions belonging to Bucl8’s extracellular portion and are specific to *B. pseudomallei* and *B. mallei*; therefore, they are less likely to have a non-specific response to commensal bacteria. Our data from Figure 2b indicates sera from mice immunized with these recombinant proteins and adjuvant recognized antigen well. This finding was further supported by the detection of antigen-specific B cells in the ELISpot assay, which indicated that a considerable number of antigen-specific B cells were generated.

As reported previously [20], the two surface-exposed loops of Bucl8 are well conserved within the *Burkholderia* clade of animal pathogens, including the majority species from the *Burkholderia cepacia* complex (BCC), *Burkholderia mallei*, *Burkholderia thailandensis* (although generally non-infectious), and *Burkholderia pseudomallei.* Thus, the β-barrel loops could be a viable vaccine target for all these species. Due to these factors in combination with the difficulty in producing endotoxin-free recombinant antigens, synthetic conjugate peptides were assessed as a valid alternative in present studies.

Our initial immunization studies indicated that the genetically inactivated diphtheria toxoid CRM_197_, used as a carrier protein, is superior to KLH in generating an antigen-specific antibody response. When combined with adjuvant, this response was augmented for all pepL1, pepL2, and Mix vaccines. Consistent with bioinformatic predictions of Bucl8 B cell epitopes and antigenicity, pepL1 elicited a more robust, consistent antibody response compared to pepL2, both when immunized individually and in mixed formulation. Binding was increased for the Mix treatment group compared to individual peptides for the various assays, indicating a possible additive effect with immunization of both peptides. Altogether, sera from mice immunized with vaccine formulation containing both peptides recognized whole Bp82 cells in two different immunoassays, indicating antigen recognition in vivo.

There are two predominant murine models for melioidosis that are utilized; C57BL/6 are more resistant to acute infection and represent a more chronic model of melioidosis, while BALB/c mice are more susceptible and represent an acute model [48]. Both strains are inbred, each with a genetically similar background that limits variability between mice. However, CD-1 mice are an outbred strain that are more genetically diverse, giving a better representation of the spread of immune responses in humans, but it is not being used for melioidosis studies. Here, we show that CD-1 and C57BL/6 mice produce similar immune responses that are TH2-biased. Both models exhibited high IgG1 titers in mice immunized with peptides and AddaVax, although the C57BL/6 mice had a titer ~10 times higher (Figure 5). Both C57BL/6 and CD-1 mice showed restricted IgG2c and IgG2a/b TH1-associated responses, respectively, which was further confirmed by muted IFN-γ levels in two different immunoassays. Hence, immunization schemes used in this study mostly resulted in a TH2-driven response. Further investigation employing T cell-specific immunoassays will be needed to better understand and characterize the type of T cell response(s) to the antigens.

Overall, we applied multiple in silico predictive analyses that identified Bucl8-derived antigens that led us to test various vaccine formulations in two different animal backgrounds. We found greater, more consistent antibody responses in mice immunized with two synthetic peptide conjugates than those with recombinant proteins. We showed that CD-1 and C57BL/6 mice had comparable immune responses. Importantly, vaccine formula containing adjuvanted peptide conjugates could also be applied against clinically relevant BCC species, ultimately reaching the goal of an effective, cross-species vaccine.

## Figures and Tables

**Table 1:** Prediction of the antigenicity, allergenicity and toxicity of Bucl8 and isolated domains.

Region	Vaxijen *^a^*	Vaxign-ML (%) *^a^*	AllergenFP v1.0 *^b^*	ToxinPred *^c^*
Bucl8	0.74	90.9	Non-allergenic	Non-Toxic
Bucl8 Nter	0.46	91.0	Non-allergenic	Non-Toxic
Bucl8 CL	2.08	90.4	Non-allergenic	Non-Toxic
Bucl8 Ct	0.85	52.0	Allergenic	Non-Toxic

**^a^** Antigenicity was computed using Vaxijen (threshold 0.4) and Vaxign-ML (protegenicity score, threshold 75%). ***^b^*** Allergenicity was computed with AllergenFP v1.0 with default settings. ***^c^*** For the toxicity, a protein scanning tool from ToxinPred was used with a threshold of 0.4. Nter; N-terminus. CL; Collagen-Like. Ct; C-terminus.

**Table 2:** Sequence based predicted B cell epitopes of Bucl8, computed using BepiPred.

Region	Sequence	Position in the Sequence
Nter	RFIRQSAKKYNRIDSSLSER	5–24
	VAPQDKQV	57–64
	AAERDAGW	77–84
	WPDNVYYGPGPLAN	148–161
	LARPKLALD	299–307
CL	GLETGRDAPHDAPAGDARRTGASGASGASRASRASRAS GASGASGASGASGASGASGASGASGAS	503–540
	SGASGASGASGASGASGASGASGASGASGASSTAGASATASASAAGHAP	573–609
Ct	ATASASAAGHAP	628–644
	ASPVAGASTPMPAAT	656–670

**Table 3:** Structure based predicted B cell epitopes of Bucl8, computed using ElliPro.

Peptide	Sequence	Position in the Bucl8 Sequence	AllergenFP 1.0	AllerTop 2.0	ToxinPred
pepL1	QHWPDNVYYGPGPLANADT	Gln146-Thr164	Non-allergenic	Non-allergenic	Non-Toxic
pepL2	GGFGVTAPFTDFLRAMNGG	Gly359-Gly377	Non-allergenic	Non-allergenic	Non-Toxic

**Table 4:** Vaccine components.

Component	Function	Description	Citation
CRM197	Conjugate	Genetically modified diphtheria toxin that is non-toxic. Has been used as a carrier protein in approved vaccines against *Haemophius influenzae type b* and several *pneumococcal* serotypes.	
KLH		A large, xenogeneic metalloprotein with multiple conjugation sites that is well-tolerated. KLH has been widely used in research and in clinical trials for cancers.	
AddaVax	Adjuvant	MF-59 like, squalene-in-oil emulsion that enhance both TH1 and TH2-like responses, augmenting the B cell memory response. MF-59 has been licensed in Europe for flu vaccines.	[36]
Quil-A		A saponin-based adjuvant that induces strong cytotoxic CD8+ response and activate both the cell-mediated and the antibody-mediated immune responses.	[37]
rBucl8-CL-Ct/ rBucl8-Ct	Antigen	Recombinantly-made proteins based on the extracellular stalk structure of Bucl8.	[19]
pepL1/pepL2		Short synthetic peptides based on the two distinct surface-exposed loops of Bucl8.	[20]

## Supplementary Materials

The following are available online at https://www.mdpi.com/article/10.3390/vaccines9111219/s1. Figure S1: Sequence-based predicted antigenic epitopes of Bucl8 by Bepipred2.0. Figure S2: Discontinuous epitope prediction via DiscoTope spanning all of Bucl8. Figure S3: Analysis of humoral responses in CD-1 mice vaccinated with KLH-conjugated pepL1. Table S1: Structure-based predicted linear epitopes by Ellipro. Table S2: MHCII binding affinity predictions of pepL1 and pepL2 using the IEDB Tool.
