# Supplementary material for: Predictive and Experimental Immunogenicity of Burkholderia Collagen-like Protein 8-Derived Antigens
Source: Vaccines (Basel). 2021 Oct 20;9(11):1219. doi: 10.3390/vaccines9111219 (PMC8621890; doi:10.3390/vaccines9111219)
Supplement: Supplementary file 1 [file vaccines-09-01219-s001.zip › vaccines-1426493-supplementary.pdf]

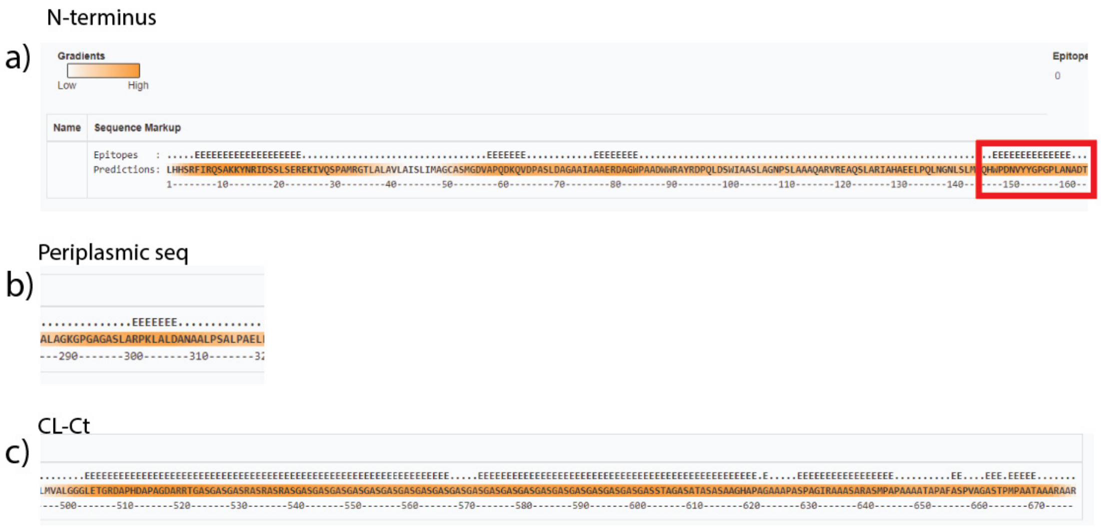

Figure S1. Sequence-based predicted antigenic epitopes of Bucl8 by Bepipred2.0. a, b, and c panels refer to N-terminal, periplasmic equatorial and CL-Ct sequences, respectively. Regions with predicted epitopes are indicated with E. Regions without predicted epitopes are not shown. The sequence of pepL1 is highlighted by the red box. Bottom numbers indicate sequence position.

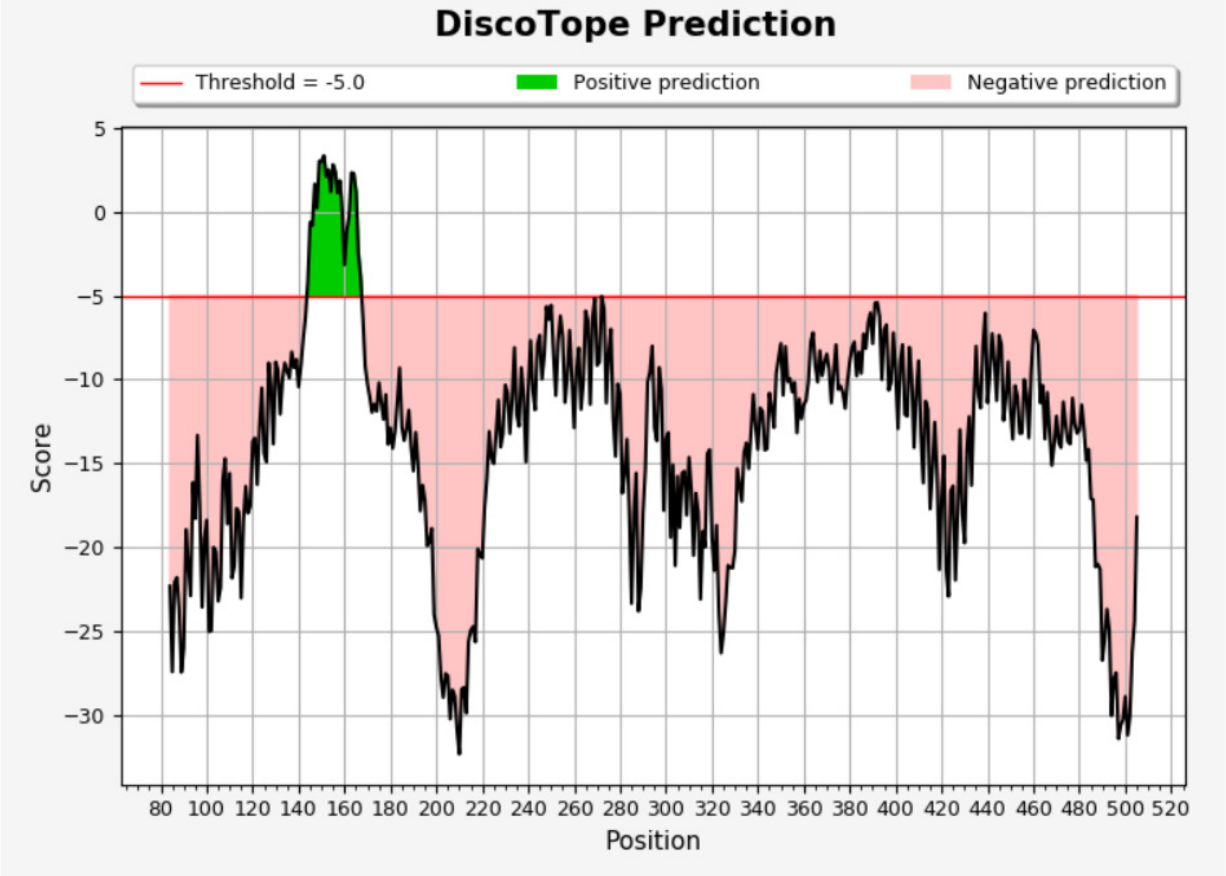

Figure S2. Discontinuous epitope prediction via DiscoTope spanning all of Bucl8. Residues 145-165, which contains the sequence for loop L1, are predicted to have a discontinuous epitope.

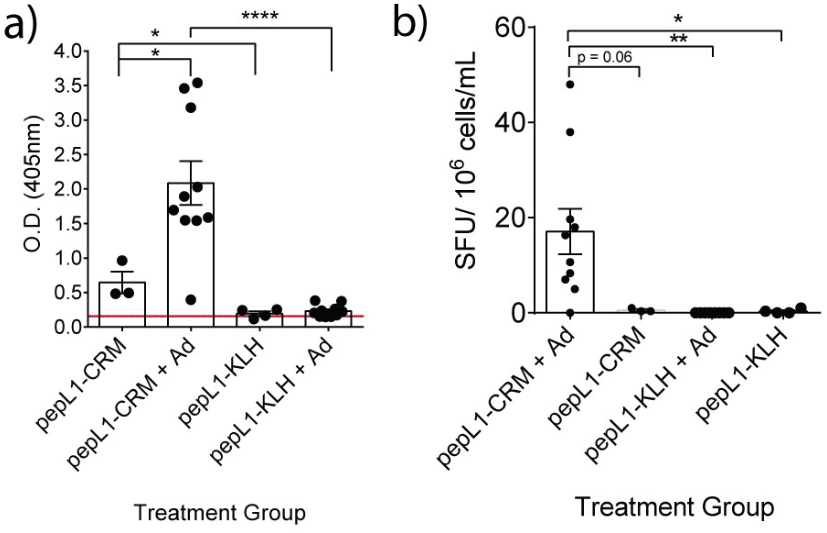

Figure S3. Analysis of humoral responses in CD-1 mice vaccinated with CRM_{197}- or KLH-conjugated pepL1. (a) Indirect ELISA with sera from mice vaccinated with pepL1-CRM_{197}- or KLH-conjugated with or without AddaVax adjuvant. Wells were coated with 1 µg of non-conjugated pepL1. Red line represents background immunoreactivity. (b) Antigen-specific B cells assessed via ELISpot. Wells were coated with 1 µg of non-conjugated pepL1 and splenocytes seeded at 10^6 cells/well. One-way ANOVA with Tukey's multiple comparison test. * $p < 0.05$, ** $p < 0.01$, **** $p < 0.0001$.

Table S1. Structure-based predicted linear epitopes by Ellipro, using the Homology model of Bucl8 generated by Modeller and the structure of the VceC efflux pump of Vibrio cholerae (PDB code 1yc9, seqid 35%) as a template.

No.	Chain	Start	End	Peptide	Number of residues	Score
1	A	139	173	GNLSLMRQHWPDNVYYGPGPLANADTWNNTGTLSL	35	0.888
2	A	233	269	YERQNALAELARKRLRAGIGTQLEVSQAEAPLPDYSR	37	0.793
3	A	341	406	IDVAKAAFYPNVDLIASLGGFAVSAPFATFLRAMNGGWSAGPALTLPIFEGGRLRAQLGVASAGYD	66	0.741
4	A	449	474	RSYDLSHAGFSRGLTDYVNVLIAQSQ	26	0.683
5	A	306	321	LDANAALPSALPAELL	16	0.627
6	A	291	299	KGPGAGASL	9	0.587

Table S2. MHCII binding affinity predictions of pepL1 (a) and pepL2 (b) using the IEDB Tool and the NN-align method. IC50 values (nM) below 100 nM are shown in orange and below 50 nM in red.

#	Peptide	Allele	Length	NN align core	NN align IC50(nM)	NN align percentile rank	NN align adjusted rank	N. of binding alleles
**a)**		**pepL1 sequence QHWPDNVYYGPGPLANADT**						
1	DNVYYGPGPLAN	HLA-DQA1*05:01/DQB1*03:01	12	VYYGPGPLA	200.5	3	8.9	3
		HLA-DRB1*01:01	12	VYYGPGPLA	344.1	31	91.92	
		HLA-DRB1*09:01	12	VYYGPGPLA	482.7	16	47.44	
2	DNVYYGPGPLANA	HLA-DQA1*05:01/DQB1*03:01	13	VYYGPGPLA	119.6	2.8	4.37	3
		HLA-DRB1*01:01	13	VYYGPGPLA	150.7	31	48.34	
		HLA-DRB1*09:01	13	VYYGPGPLA	396.9	20	31.19	
3	DNVYYGPGPLANAD	HLA-DQA1*05:01/DQB1*03:01	14	VYYGPGPLA	92.5	3.5	3.77	3
		HLA-DRB1*01:01	14	VYYGPGPLA	148.4	36	38.76	
		HLA-DRB1*09:01	14	VYYGPGPLA	417.7	27	29.07	
4	DNVYYGPGPLANADT	HLA-DQA1*05:01/DQB1*03:01	15	VYYGPGPLA	71.5	4.4	4.4	3
		HLA-DRB1*01:01	15	VYYGPGPLA	213.2	39	39	
		HLA-DRB1*09:01	15	VYYGPGPLA	464.7	33	33	
5	HWPDNVYYGPGPLA	HLA-DQA1*05:01/DQB1*03:01	14	VYYGPGPLA	311.8	12	12.92	2
		HLA-DRB1*09:01	14	NVYYGPGPL	425.9	27	29.07	
6	HWPDNVYYGPGPLAN	HLA-DQA1*05:01/DQB1*03:01	15	VYYGPGPLA	104.8	6.6	6.6	2
		HLA-DRB1*09:01	15	NVYYGPGPL	383.7	30	30	
7	HWPDNVYYGPGPLANA	HLA-DQA1*05:01/DQB1*03:01	16	VYYGPGPLA	58.1	5.3	6.12	2
		HLA-DRB1*09:01	16	NVYYGPGPL	354.9	31	35.78	
8	HWPDNVYYGPGPLANAD	HLA-DQA1*05:01/DQB1*03:01	17	VYYGPGPLA	45.2	5.4	9.2	2
		HLA-DRB1*09:01	17	NVYYGPGPL	380.7	36	61.32	
9	HWPDNVYYGPGPLANADT	HLA-DRB1*09:01	18	NVYYGPGPL	386.2	41	121.35	1
10	NVYYGPGPLANA	HLA-DQA1*05:01/DQB1*03:01	12	VYYGPGPLA	147.6	1.9	5.63	2
		HLA-DRB1*01:01	12	VYYGPGPLA	192.4	22	65.24	
11	NVYYGPGPLANAD	HLA-DQA1*05:01/DQB1*03:01	13	VYYGPGPLA	123.4	3	4.68	2
		HLA-DRB1*01:01	13	VYYGPGPLA	153.5	31	48.34	
12	NVYYGPGPLANADT	HLA-DQA1*05:01/DQB1*03:01	14	VYYGPGPLA	102.2	4	4.31	2
		HLA-DRB1*01:01	14	VYYGPGPLA	156.5	37	39.84	
13	PDNVYYGPGPLA	HLA-DRB1*09:01	12	NVYYGPGPL	438.7	15	44.48	2
		HLA-DQA1*05:01/DQB1*03:01	12	VYYGPGPLA	482.7	7.2	21.35	

14	PDNVYYGPGPLAN	HLA-DQA1*05:01/DQB1*03:01	13	VYYGPGPLA	176.1	4.5	7.02	3
		HLA-DRB1*01:01	13	VYYGPGPLA	301.2	43	67.05	
		HLA-DRB1*09:01	13	VYYGPGPLA	348.5	18	28.07	
15	PDNVYYGPGPLANA	HLA-DQA1*05:01/DQB1*03:01	14	VYYGPGPLA	98.7	3.8	4.09	3
		HLA-DRB1*01:01	14	VYYGPGPLA	164.5	38	40.92	
		HLA-DRB1*09:01	14	VYYGPGPLA	302.9	22	23.69	
16	PDNVYYGPGPLANAD	HLA-DQA1*05:01/DQB1*03:01	15	VYYGPGPLA	70.3	4.3	4.3	3
		HLA-DRB1*01:01	15	VYYGPGPLA	227	40	40	
		HLA-DRB1*09:01	15	VYYGPGPLA	341.1	27	27	
17	PDNVYYGPGPLANADT	HLA-DQA1*05:01/DQB1*03:01	16	VYYGPGPLA	55.5	5	5.77	3
		HLA-DRB1*01:01	16	VYYGPGPLA	382	40	46.17	
		HLA-DRB1*09:01	16	NVYYGPGPL	382.8	33	38.09	
18	QHWPDNVYYGPGPLA	HLA-DQA1*05:01/DQB1*03:01	15	VYYGPGPLA	244.6	14	14	2
		HLA-DRB1*09:01	15	NVYYGPGPL	468.8	33	33	
19	QHWPDNVYYGPGPLAN	HLA-DQA1*05:01/DQB1*03:01	16	VYYGPGPLA	83.9	7.9	9.12	2
		HLA-DRB1*09:01	16	NVYYGPGPL	424.2	35	40.4	
20	QHWPDNVYYGPGPLANA	HLA-DQA1*05:01/DQB1*03:01	17	VYYGPGPLA	50.5	6.2	10.56	2
		HLA-DRB1*09:01	17	NVYYGPGPL	374.2	36	61.32	
21	QHWPDNVYYGPGPLANAD	HLA-DQA1*05:01/DQB1*03:01	18	VYYGPGPLA	42	6.1	18.05	2
		HLA-DRB1*09:01	18	NVYYGPGPL	380.1	40	118.39	
22	VYYGPGPLANAD	HLA-DQA1*05:01/DQB1*03:01	12	VYYGPGPLA	221	3.3	9.79	2
		HLA-DRB1*01:01	12	VYYGPGPLA	386.6	33	97.86	
23	VYYGPGPLANADT	HLA-DQA1*05:01/DQB1*03:01	13	VYYGPGPLA	196.5	5	7.8	2
		HLA-DRB1*01:01	13	VYYGPGPLA	313.7	44	68.61	
24	WPDNVYYGPGPLA	HLA-DQA1*05:01/DQB1*03:01	13	VYYGPGPLA	379.5	9.1	14.19	2
		HLA-DRB1*09:01	13	NVYYGPGPL	419.6	21	32.75	
25	WPDNVYYGPGPLAN	HLA-DQA1*05:01/DQB1*03:01	14	VYYGPGPLA	137	5.6	6.03	3
		HLA-DRB1*09:01	14	VYYGPGPLA	353.2	24	25.84	
		HLA-DRB1*01:01	14	VYYGPGPLA	379.8	54	58.15	
26	WPDNVYYGPGPLANA	HLA-DQA1*05:01/DQB1*03:01	15	VYYGPGPLA	74.4	4.6	4.6	3
		HLA-DRB1*01:01	15	VYYGPGPLA	288.3	44	44	
		HLA-DRB1*09:01	15	VYYGPGPLA	325.6	27	27	
27	WPDNVYYGPGPLANAD	HLA-DQA1*05:01/DQB1*03:01	16	VYYGPGPLA	53.3	4.9	5.66	3

		HLA-DRB1*09:01	16	VYYGPGPLA	367	31	35.78	
		HLA-DRB1*01:01	16	VYYGPGPLA	455.4	43	49.63	
28	WPDNVYYGPGPLANADT	HLA-DQA1*05:01/DQB1*03:01	17	VYYGPGPLA	45.5	5.4	9.2	2
		HLA-DRB1*09:01	17	NVYYGPGPL	393.1	37	63.02	

b) pepL2 sequence GGFGVTAPFTDFLRAMNGG

	Peptide	Allele	Length	NN align core	NN align IC50(nM)	NN align percentile rank	NN align adjusted rank	N.of binding alleles
1	APFTDFLRAMNG	HLA-DRB1*11:01	12	FTDFLRAMN	151.2	5.8	17.2	2
		HLA-DRB1*01:01	12	TDFLRAMNG	245.9	26	77.1	
2	APFTDFLRAMNGG	HLA-DRB1*11:01	13	FTDFLRAMN	90.8	6.4	9.98	2
		HLA-DRB1*01:01	13	TDFLRAMNG	105	25	38.98	
3	FGVTAPFTDFLRA	HLA-DPA1*01:03/DPB1*02:01	13	APFTDFLRA	392.6	9.6	14.97	1
4	FGVTAPFTDFLRAM	HLA-DPA1*01:03/DPB1*02:01	14	APFTDFLRA	257.2	8.9	9.58	1
5	FGVTAPFTDFLRAMN	HLA-DPA1*01:03/DPB1*02:01	15	APFTDFLRA	254.4	11	11	2
		HLA-DPA1*03:01/DPB1*04:02	15	TAPFTDFLR	400.6	17	17	
6	FGVTAPFTDFLRAMNG	HLA-DPA1*01:03/DPB1*02:01	16	APFTDFLRA	285.3	14	16.16	5
		HLA-DRB1*11:01	16	FTDFLRAMN	313.4	18	20.78	
		HLA-DPA1*02:01/DPB1*05:01	16	TAPFTDFLR	323.4	12	13.85	
		HLA-DPA1*03:01/DPB1*04:02	16	TAPFTDFLR	379.9	23	26.55	
		HLA-DRB1*08:02	16	FTDFLRAMN	434.7	23	26.55	
7	FGVTAPFTDFLRAMNGG	HLA-DPA1*02:01/DPB1*05:01	17	TAPFTDFLR	206.6	14	23.85	5
		HLA-DRB1*11:01	17	FTDFLRAMN	272.3	16	27.25	
		HLA-DRB1*08:02	17	FTDFLRAMN	275.6	22	37.47	
		HLA-DPA1*01:03/DPB1*02:01	17	APFTDFLRA	311.5	16	27.25	
		HLA-DPA1*03:01/DPB1*04:02	17	TAPFTDFLR	385.3	29	49.39	
8	GFGVTAPFTDFL	HLA-DRB1*07:01	12	FGVTAPFTD	443.5	21	62.27	2
		HLA-DQA1*05:01/DQB1*02:01	12	VTAPFTDFL	487.6	4.4	13.05	
9	GFGVTAPFTDFLR	HLA-DRB1*09:01	13	FGVTAPFTD	483.8	23	35.87	1
10	GFGVTAPFTDFLRA	HLA-DPA1*01:03/DPB1*02:01	14	APFTDFLRA	346.7	12	12.92	1
11	GFGVTAPFTDFLRAM	HLA-DPA1*01:03/DPB1*02:01	15	APFTDFLRA	241.7	11	11	1
12	GFGVTAPFTDFLRAMN	HLA-DPA1*01:03/DPB1*02:01	16	APFTDFLRA	241.5	12	13.85	3
		HLA-DPA1*02:01/DPB1*05:01	16	TAPFTDFLR	305.8	11	12.7	
		HLA-DPA1*03:01/DPB1*04:02	16	TAPFTDFLR	309.1	20	23.09	
13	GFGVTAPFTDFLRAMNG	HLA-DPA1*02:01/DPB1*05:01	17	TAPFTDFLR	181.9	13	22.14	6
		HLA-DPA1*01:03/DPB1*02:01	17	APFTDFLRA	260	14	23.85	
		HLA-DRB1*08:02	17	FTDFLRAMN	291.8	23	39.17	
		HLA-DPA1*03:01/DPB1*04:02	17	TAPFTDFLR	307.7	25	42.58	
		HLA-DRB1*11:01	17	FTDFLRAMN	369.6	19	32.36	

		HLA-DQA1*05:01/DQB1*02:01	17	VTAPFTDFL	483.5	18	30.66	
14	GFGVTAPFTDFLRAMNGG	HLA-DPA1*02:01/DPB1*05:01	18	TAPFTDFLR	145.3	15	44.4	6
		HLA-DRB1*08:02	18	FTDFLRAMN	202.5	22	65.11	
		HLA-DPA1*01:03/DPB1*02:01	18	APFTDFLRA	273.4	17	50.31	
		HLA-DRB1*11:01	18	FTDFLRAMN	285.1	17	50.31	
		HLA-DPA1*03:01/DPB1*04:02	18	TAPFTDFLR	325.4	31	91.75	
		HLA-DQA1*05:01/DQB1*02:01	18	VTAPFTDFL	496.5	21	62.15	
15	GGFGVTAPFTDF	HLA-DRB1*07:01	12	FGVTAPFTD	482.7	22	65.24	1
16	GGFGVTAPFTDFL	HLA-DQA1*05:01/DQB1*02:01	13	GFGVTAPFT	331.7	4.6	7.17	3
		HLA-DRB1*09:01	13	FGVTAPFTD	333.8	18	28.07	
		HLA-DRB1*07:01	13	FGVTAPFTD	383.2	25	38.98	
17	GGFGVTAPFTDFLR	HLA-DRB1*09:01	14	FGVTAPFTD	334.3	23	24.77	4
		HLA-DQA1*05:01/DQB1*02:01	14	GFGVTAPFT	370	7.5	8.08	
		HLA-DRB1*07:01	14	FGVTAPFTD	486.9	32	34.46	
		HLA-DPA1*01:03/DPB1*02:01	14	GVTAPFTDF	499.4	15	16.15	
18	GGFGVTAPFTDFLRA	HLA-DPA1*01:03/DPB1*02:01	15	APFTDFLRA	341.9	14	14	3
		HLA-DQA1*05:01/DQB1*02:01	15	GFGVTAPFT	375.7	9.8	9.8	
		HLA-DRB1*09:01	15	FGVTAPFTD	393.8	30	30	
19	GGFGVTAPFTDFLRAM	HLA-DPA1*01:03/DPB1*02:01	16	APFTDFLRA	243	12	13.85	5
		HLA-DQA1*05:01/DQB1*02:01	16	GFGVTAPFT	360.2	12	13.85	
		HLA-DRB1*09:01	16	FGVTAPFTD	459.8	36	41.55	
		HLA-DQA1*05:01/DQB1*03:01	16	FGVTAPFTD	469.2	29	33.47	
		HLA-DPA1*03:01/DPB1*04:02	16	TAPFTDFLR	489.4	27	31.17	
20	GGFGVTAPFTDFLRAMN	HLA-DPA1*02:01/DPB1*05:01	17	TAPFTDFLR	183.1	13	22.14	7
		HLA-DPA1*01:03/DPB1*02:01	17	APFTDFLRA	237.7	13	22.14	
		HLA-DPA1*03:01/DPB1*04:02	17	TAPFTDFLR	276.5	23	39.17	
		HLA-DQA1*05:01/DQB1*02:01	17	GFGVTAPFT	369.4	14	23.85	
		HLA-DQA1*05:01/DQB1*03:01	17	FGVTAPFTD	405.3	33	56.21	
		HLA-DRB1*08:02	17	FTDFLRAMN	426.9	32	54.5	
		HLA-DRB1*09:01	17	FGVTAPFTD	447.7	39	66.43	
21	GGFGVTAPFTDFLRAMNG	HLA-DPA1*02:01/DPB1*05:01	18	TAPFTDFLR	138.1	15	44.4	8
		HLA-DRB1*08:02	18	FTDFLRAMN	243	26	76.95	
		HLA-DPA1*01:03/DPB1*02:01	18	APFTDFLRA	246.9	15	44.4	
		HLA-DPA1*03:01/DPB1*04:02	18	TAPFTDFLR	288	29	85.83	
		HLA-DQA1*05:01/DQB1*02:01	18	GFGVTAPFT	357.6	16	47.35	
		HLA-DQA1*05:01/DQB1*03:01	18	FGVTAPFTD	376.3	36	106.55	
		HLA-DRB1*11:01	18	FTDFLRAMN	388.3	21	62.15	

		HLA-DRB1*09:01	18	FGVTAPFTD	466	45	133.19	
22	GVTAPFTDFLRAM	HLA-DPA1*01:03/DPB1*02:01	13	APFTDFLRA	328.4	8.5	13.25	1
23	GVTAPFTDFLRAMN	HLA-DPA1*01:03/DPB1*02:01	14	APFTDFLRA	307.7	11	11.84	2
		HLA-DRB1*11:01	14	FTDFLRAMN	461	23	24.77	
24	GVTAPFTDFLRAMNG	HLA-DRB1*11:01	15	FTDFLRAMN	212.6	16	16	2
		HLA-DPA1*01:03/DPB1*02:01	15	APFTDFLRA	344	14	14	
25	GVTAPFTDFLRAMNGG	HLA-DRB1*11:01	16	FTDFLRAMN	201.8	14	16.16	5
		HLA-DRB1*08:02	16	FTDFLRAMN	312.5	17	19.62	
		HLA-DPA1*01:03/DPB1*02:01	16	APFTDFLRA	387.9	17	19.62	
		HLA-DPA1*02:01/DPB1*05:01	16	TAPFTDFLR	444.3	15	17.31	
		HLA-DPA1*03:01/DPB1*04:02	16	TAPFTDFLR	499.8	27	31.17	
26	PFTDFLRAMNGG	HLA-DRB1*11:01	12	FTDFLRAMN	127.1	5	14.83	2
		HLA-DRB1*01:01	12	TDFLRAMNG	129.8	17	50.41	
27	TAPFTDFLRAMN	HLA-DRB1*11:01	12	FTDFLRAMN	468.2	13	38.55	1
28	TAPFTDFLRAMNG	HLA-DRB1*11:01	13	FTDFLRAMN	139.2	8.7	13.57	2
		HLA-DRB1*01:01	13	TDFLRAMNG	241.2	39	60.82	
29	TAPFTDFLRAMNGG	HLA-DRB1*11:01	14	FTDFLRAMN	96.6	8.7	9.37	2
		HLA-DRB1*01:01	14	TDFLRAMNG	156.7	37	39.84	
30	VTAPFTDFLRAM	HLA-DPA1*01:03/DPB1*02:01	12	APFTDFLRA	418.3	7.2	21.35	1
31	VTAPFTDFLRAMN	HLA-DPA1*01:03/DPB1*02:01	13	APFTDFLRA	377	9.4	14.66	2
		HLA-DRB1*11:01	13	FTDFLRAMN	441.7	19	29.63	
32	VTAPFTDFLRAMNG	HLA-DRB1*11:01	14	FTDFLRAMN	155.6	13	14	3
		HLA-DRB1*01:01	14	TDFLRAMNG	344.4	52	55.99	
		HLA-DPA1*01:03/DPB1*02:01	14	APFTDFLRA	413.6	13	14	
33	VTAPFTDFLRAMNGG	HLA-DRB1*11:01	15	FTDFLRAMN	131.2	11	11	4
		HLA-DRB1*08:02	15	FTDFLRAMN	393.3	13	13	
		HLA-DRB1*01:01	15	TDFLRAMNG	426.6	52	52	
		HLA-DPA1*01:03/DPB1*02:01	15	APFTDFLRA	468	17	17	
